# Dysphagia Unmasked: A Case Report of Esophageal Leiomyomatosis

**DOI:** 10.7759/cureus.48158

**Published:** 2023-11-02

**Authors:** Lilian M Haji, Husain H Faraj, Bader N Abdulaziz, Hissa A Alaradi, Ahlam Alharbi

**Affiliations:** 1 General Practice, First Moscow State Medical University, Moscow, RUS; 2 General Practice, Southeast University, Nanjing, CHN; 3 Family Medicine, Primary Health Care Center, Riyadh, SAU; 4 Family Medicine, Jordan University of Science and Technology, Irbid, JOR

**Keywords:** case report, magnetic resonance imaging, computed tomography, endoscopy, esophageal leiomyomatosis, dysphagia

## Abstract

Esophageal leiomyomatosis, an uncommon benign condition marked by the proliferation of smooth muscle cells within the esophageal wall, frequently presents diagnostic challenges due to its rarity and diverse clinical manifestations. In this case report, we document the clinical journey of a 28-year-old female who presented with a two-year history of progressive dysphagia. Upon physical examination and endoscopy, a submucosal mass in the lower esophagus was identified, prompting further imaging and subsequent biopsy, which confirmed the diagnosis of leiomyomatosis. A multidisciplinary team recommended surgical intervention, leading to a minimally invasive laparoscopic resection of the esophageal leiomyomas. Postoperatively, the patient experienced a substantial improvement in her dysphagia and was discharged in stable condition. This case not only underscores the importance of a multidisciplinary approach in achieving an accurate diagnosis but also highlights the successful application of minimally invasive surgical techniques for alleviating symptoms in esophageal leiomyomatosis patients. The rarity and varied clinical presentation of this condition emphasize the need for individualized and tailored management strategies.

## Introduction

Esophageal leiomyomatosis is a rare benign condition characterized by the proliferation of smooth muscle cells within the esophageal wall, leading to the development of multiple leiomyomas [1]. While benign in nature, this disorder can present with debilitating symptoms, such as progressive dysphagia, regurgitation, and discomfort during meals, significantly impacting the patient's quality of life [2]. Although leiomyomatosis predominantly affects the uterine and gastrointestinal tracts, its occurrence in the esophagus remains exceedingly uncommon [3]. The pathogenesis of esophageal leiomyomatosis is not yet fully elucidated. Current understanding suggests that genetic mutations and hormonal factors may contribute to the development of these leiomyomas. This rarity, along with its diverse clinical presentation, often poses diagnostic challenges, requiring a comprehensive approach that encompasses clinical evaluation, endoscopy, histopathological analysis, and advanced imaging modalities [1]. This case report aims to present the clinical journey of a young woman who, after years of persistent dysphagia and discomfort, was diagnosed with esophageal leiomyomatosis. This case not only emphasizes the clinical and diagnostic complexities associated with this rare condition but also underscores the importance of a multidisciplinary approach in providing an accurate diagnosis and tailored therapeutic management. Furthermore, the case highlights the successful application of minimally invasive surgical techniques to provide symptomatic relief and improve the patient's overall well-being.

## Case presentation

A 28-year-old Saudi female presented to our gastroenterology clinic with a chief complaint of progressive dysphagia, primarily for solids, for the past two years. She reported a sensation of food getting stuck in her chest, which was associated with occasional regurgitation and discomfort during meals. Her medical history was unremarkable, and she denied any significant weight loss, heartburn, or gastrointestinal bleeding. Family history was non-contributory for gastrointestinal conditions.

Upon physical examination, the patient appeared well nourished and in no acute distress. Vital signs were stable, and there were no signs of anemia or lymphadenopathy. Notably, there were no stigmata of chronic liver disease or other systemic illnesses. Examination of the oral cavity revealed no abnormalities, and no palpable masses were detected in the neck or supraclavicular regions.

Further investigation was initiated, which included an upper gastrointestinal endoscopy. The procedure revealed a submucosal mass in the lower esophagus, approximately 25 cm from the incisors. The overlying mucosa appeared normal, and biopsies were obtained.

**Figure 1:**
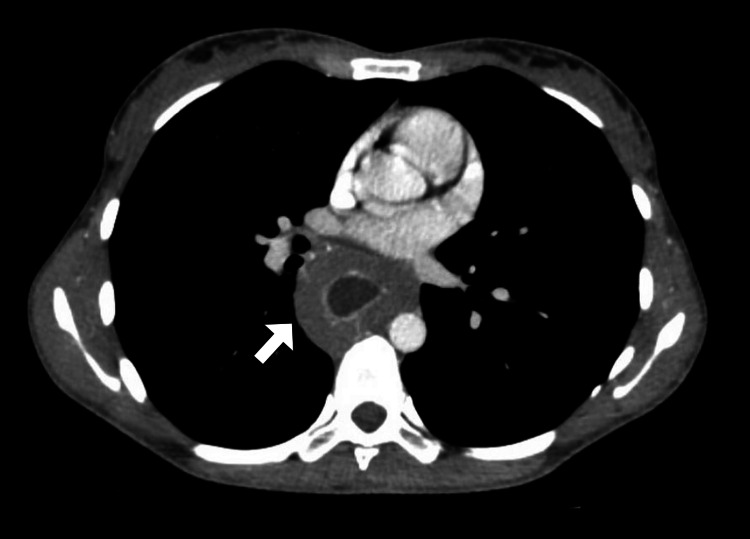
Axial CT image of the chest illustrating diffuse circumferential wall thickening of the esophagus (arrow). CT: computed tomography

**Figure 2:**
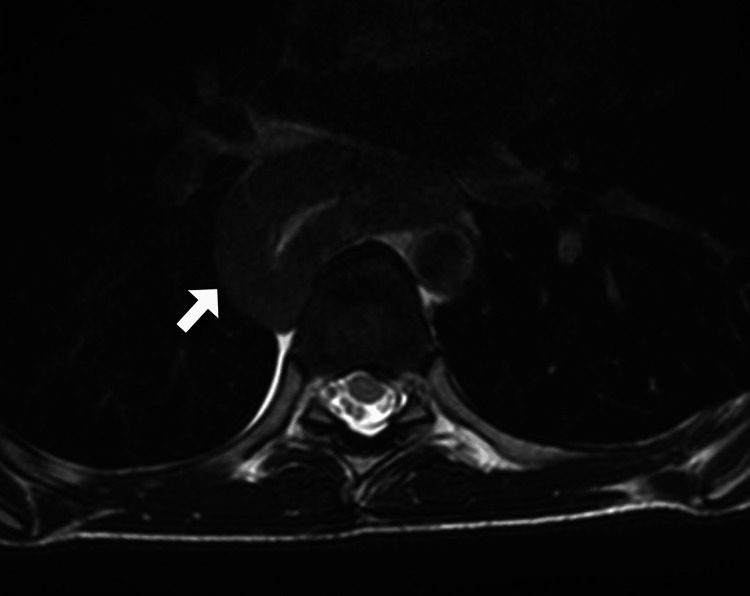
Axial T2-weighted MR image of the chest revealing diffuse circumferential wall thickening of the esophagus (arrow). MR: magnetic resonance

In order to evaluate the extent and characteristics of the lesion, the patient underwent additional imaging studies. A contrast-enhanced computed tomography scan of the chest revealed diffuse circumferential wall thickening of the esophagus with luminal narrowing and proximal dilatation of the esophagus (Figure 1). No evidence of lymph node involvement or distant metastasis was observed. Subsequently, magnetic resonance imaging was also performed, which confirmed the findings in line with the computed tomography (Figure 2).

**Figure 3:**
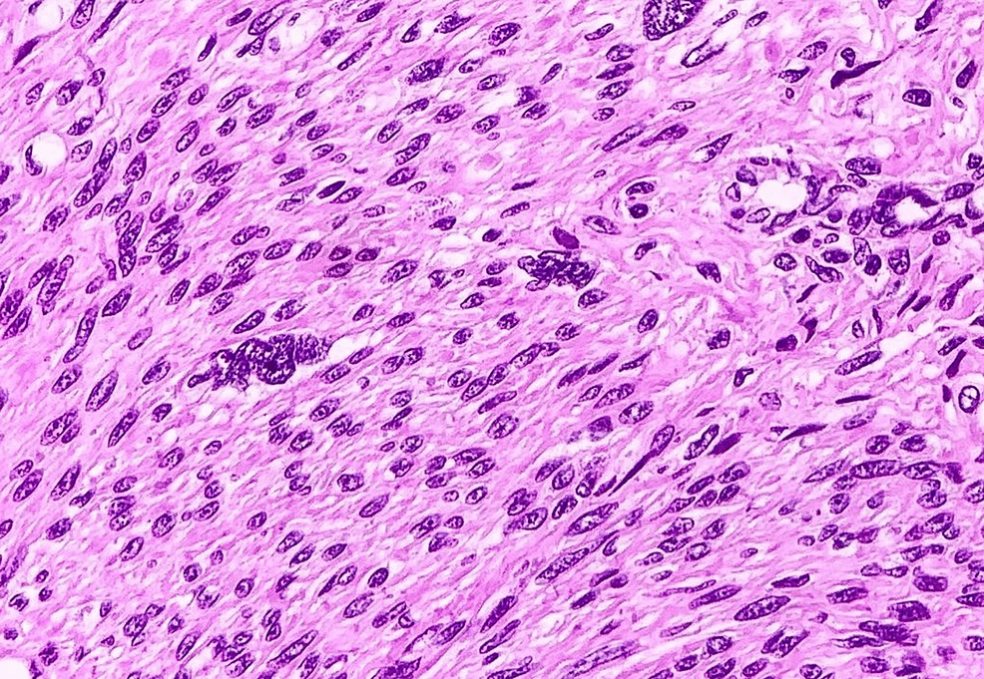
Histopathological image demonstrating spindle-shaped cells consistent with leiomyoma.

The differential diagnoses considered included leiomyomatosis, gastrointestinal stromal tumor, leiomyosarcoma, and esophageal duplication cyst. However, histopathological examination of the biopsy specimens showed spindle-shaped cells with elongated nuclei, consistent with leiomyomatosis (Figure 3).

Given the extent and symptomatic nature of the patient's condition, a multidisciplinary team discussion was conducted, and the decision was made to proceed with surgical intervention. The patient underwent a minimally invasive laparoscopic resection of the lower esophageal leiomyomas. The patient's postoperative recovery was uneventful. She was discharged on the fifth postoperative day, and in the subsequent follow-up visits, she reported a significant improvement in her dysphagia, with the ability to comfortably tolerate a regular diet. The patient continued to have regular follow-up visits to monitor her recovery progress and to assess for any signs of recurrence. In the follow-up period of one year, the patient had no symptoms of recurrence.

## Discussion

Esophageal leiomyomatosis is a rare benign condition characterized by the proliferation of smooth muscle cells in the esophagus, leading to esophageal wall thickening. This case report highlights a young woman with progressive dysphagia, ultimately diagnosed with this rare condition. Esophageal leiomyomatosis constitutes less than 1% of esophageal neoplasms, often posing diagnostic challenges as it is frequently misdiagnosed as more common esophageal disorders, such as achalasia or gastroesophageal reflux disease [1,4]. Therefore, maintaining clinical suspicion, thorough evaluation, and including this condition in the differential diagnosis of dysphagia are crucial for early detection and accurate management.

The most common symptom of esophageal leiomyomatosis is dysphagia, which our patient predominantly experienced. The gradual onset of dysphagia can result in delayed diagnosis, initially attributed to conditions, such as gastroesophageal reflux disease or achalasia [3,5]. This underscores the need for comprehensive clinical evaluation and considering rare causes in unexplained dysphagia cases.

Imaging plays a significant role in assessing esophageal leiomyomatosis. In our case, the findings were consistent with previously reported cases, showing well-circumscribed intramural esophageal masses [6-8]. These imaging techniques aid in surgical planning and treatment decisions.

The management of esophageal leiomyomatosis depends on various factors, including lesion size, location, and symptom severity [4]. As discussed in the literature, asymptomatic patients with small lesions may require clinical and endoscopic follow-up. By contrast, symptomatic patients, such as our case, and those with larger lesions are candidates for surgical intervention [1,2].

Surgical resection, particularly enucleation of tumors, is the primary approach for larger lesions [4,5]. In our case, a minimally invasive laparoscopic approach was chosen, aligning with the trend in modern surgery to minimize invasiveness and promote faster recovery. This decision highlights the adaptability of surgical techniques and individualized patient care. Myomectomy is rarely performed due to reported unsatisfactory outcomes. Total esophagectomy is reserved for patients with severe and progressive dysphagia, emphasizing the importance of tailored management in this rare condition [2,3].

## Conclusions

The presented case of esophageal leiomyomatosis in a young woman with persistent dysphagia serves as a poignant reminder of the complexity and challenges associated with this rare condition. Through a multidisciplinary approach that encompassed clinical evaluation, endoscopy, and advanced imaging, we achieved an accurate diagnosis, which is crucial in the management of this disorder. The successful application of minimally invasive surgical techniques not only provided symptomatic relief but also highlighted the evolving trends in surgical approaches for esophageal leiomyomatosis. This case underscores the significance of heightened clinical suspicion and thorough evaluation when faced with unexplained dysphagia, as early diagnosis can lead to better outcomes and improved patient quality of life. Furthermore, sharing such cases and experiences through case reports and research contributes to our collective understanding of esophageal leiomyomatosis and reinforces the importance of individualized treatment strategies.
